# Lightweight Insulation Boards Based on Lignocellulosic Particles Glued with Agents of Natural Origin

**DOI:** 10.3390/ma14123219

**Published:** 2021-06-10

**Authors:** Radosław Mirski, Dorota Dziurka, Marcin Kuliński, Adam Derkowski

**Affiliations:** Department of Wood-Based Materials, Poznań University of Life Sciences, 60-627 Poznań, Poland; dorota.dziurka@up.poznan.pl (D.D.); marcin.kulinski@up.poznan.pl (M.K.); adam.derkowski@up.poznan.pl (A.D.)

**Keywords:** lightweight insulation boards, natural-origin adhesives, wood fibers, fiberboard, starch

## Abstract

In this study, the possibility of using adhesives of natural origin for the manufacture of wood fiber-based lightweight panels was investigated. The boards, of a density ranging from 150 to 250 kg/m^3^, were glued together using commercial urea–formaldehyde resin (control board), solutions of rye flour and potato starch and two types of starch: oxidized and gelatinized. The density and density profile, compressive strength, modulus of elasticity, acoustic properties and thermal conductivity were determined in the produced boards. These studies show that when food components are used as binding agents in the manufacture of lightweight wood fiberboards, the properties obtained can be comparable with those of commercial boards manufactured using synthetic agents.

## 1. Introduction

Properties of wood-based panels to a significant extent depend on the bonding agent used in their manufacture. In the production of building materials, synthetic resins, primarily formaldehyde resins, i.e., phenol–formaldehyde (PF), phenol–resorcinol–formaldehyde (PRF), melamine–urea–formaldehyde (MUF), urea–formaldehyde (UF) and isocyanine resins, have been applied for years. The advantages of formaldehyde resins are connected with their relatively low price and the good physicomechanical properties of materials manufactured using these resins [1,2,3,4,5,6,7,8,9,10]. Moreover, boards bonded with PF resin are characterized by low free formaldehyde and phenol emissions both during the manufacturing process and during their service life, thanks to which they are classified as emission class E0 and work stations involved in their production meet higher occupational safety standards [11] compared, e.g., to the use of urea–formaldehyde resins. Free formaldehyde emissions are dependent both on the quantity and quality of used resins [12,13,14,15].

Construction boards are not only medium- or high-density boards, i.e., structural panels, but also lightweight panels, i.e., those of low and very low density dedicated to thermal and acoustic insulation [16,17,18]. These panels are typically manufactured from engineered wood fibers. In Europe, Steico is the leading company producing wood fiber insulation panels used to insulate building structures. Its products are manufactured using isocyanate adhesives (polymeric diphenyl methane diisocyanate (pMDI)), and their density ranges from 100 to 250 kg/m^3^. A reduction in density results in an almost proportional decrease in the amount of used resin [19]. However, irrespective of the quantity and type of the applied bonding agent, the panels are not environmentally friendly. In turn, in recent years we have been observing a trend to eliminate materials for which recycling is problematic or that are not biodegradable. Consequently, as has been observed since at least the 1980s, new engineered materials are being searched for, in line with the concept of sustainable development. More eco-friendly materials are at present primarily at the research and development (R&D) stage; as a result, their prices are much higher than those of commercially produced materials. A certain alternative to synthetic compounds based on formaldehyde may be provided by their replacement with more environmentally friendly agents [20,21,22,23]. Another alternative is offered by panels manufactured without the application of an adhesive. However, except for the older wet fiberboard forming systems, the current adhesive-free wood bonding systems have not been implemented on a broader scale due to the required application of high pressure and high pressing temperature [24,25]. Natural-origin adhesives are an alternative to such solutions. Natural origin adhesives may include starch, sucrose, glucose, chitosan, lignin, tannin, protein, gums and citric acid and have been used in numerous studies [26,27,28,29,30,31,32,33,34,35,36,37,38,39,40,41]. Application of adhesives based on natural components gives satisfactory results in terms of mechanical strength properties, while inferior properties are observed in terms of water soaking or swelling in thickness [42,43,44]. Since most cited studies concern tests conducted on plywood or particleboards of densities exceeding 600 kg/m^3^, swelling in thickness may be crucial in this case. However, this parameter is less significant in the case of lightweight panels dedicated to thermal or acoustic insulation. Thus, the aim of this study was to evaluate the applicability of compounds or products containing starch in the manufacture of lightweight panels from wood fibers.

## 2. Materials and Methods

Under laboratory conditions, panels were manufactured from pine fibers typically used in the manufacture of dry-formed fiberboards. Panels were bonded using a commercial urea–formaldehyde resin (UF), rye flour and potato starch solutions and two starch types: E 1404 (oxidized starch) and gelatinized starch. Basic properties of UF resin (Silekol Sp. z o.o., Kędzierzyn-Koźle, Poland) used in the tests were as follows: viscosity, 650 mPa·s; solid content, 69%; gel time at 100 °C, 69 s; density, 1.282 g/cm^3^; pH, 8.09.

The rye flour solution was prepared from 100 g water and 25 g rye flour of approx. 3.5% moisture content. The solution was mixed until a homogeneous blend was obtained; it was left for 20 min and after being mixed again it was used as an adhesive. In turn, the potato starch solution was prepared as follows: 4 g of starch was added to 100 g of water. After being thoroughly mixed to produce a suspension, the solution was heated while mixing continuously to ensure its thickening. Fibers of 7.2% moisture content were coated with bonding agent solutions at 12% adhesive dry mass for UF resin, 12% and 6% adhesive dry mass for the rye flour solution and 2.4% and 3.6% adhesive dry mass for the potato starch solution.

In turn, starch was applied onto fibers with approx. 12% and 28% moisture content. Three days before the bonding process, fibers of a 7.2% moisture content were sprayed with water in an amount ensuring an increase in the fiber moisture content to 15% and 30%. After thorough mixing, 0.5 kg batches were placed in a double-sided plastic bag and sealed tightly. At least twice daily, the mass in the bags was mixed thoroughly. The fiber moisture content prior to bonding amounted to approx. 12% (± 0.2%) and 28% (± 0.34%). Starch was applied in the amount ensuring a bonding rate at 12% or 20%. Starch was applied using a pneumatic lacquer spray gun. The mat was formed from such prepared material. After formation, the mat was pressed at 0.8 MPa at the temperature of heat press platens of 180 °C (semi-automatic press with the dimensions of the shelves 80 cm × 60 cm, Siempelkamp). Panels were manufactured at a thickness of 25 mm and design density ranging from 150 to 250 kg/m^3^. Pressing time was dependent on the type of the applied bonding agent. More detailed data are given in Table 1. For each panel type, three panels were manufactured, formed from frames of 20 cm × 40 cm.

One of the panels was assigned only for the determination of thermal conductivity, with the panel being dried to constant mass at a temperature of 103 °C, while the other two were used in the other tests and conditioned at 20 ± 2 °C and relative humidity of 60 ± 5%. After the conditioning period (14 days), panels with approx. 7.4–8.2% moisture content the following parameters were assessed in terms of the following:

- Density (ρ) based on PN-EN 323:1999 [45].
- Compressive strength (f_v_)—the value of compression stress at 10% true strain according to PN-EN 826 [46].
- Modulus of elasticity (E) according to PN-EN 310 [47].
- Thermal conductivity was determined using a measuring system presented in a study by Mirski [48]. In this case, thermal conductivity λ was calculated from Equation (1):
  λ = q × d/(Tg − Tc) [W/mK] (1)
  where q is the heat flux density calculated from Equation (2):
  q = C × U [W/m^2^] (2)
  where C is the sensor calibration factor of 35.8 (W/ m^2^ mV), U is the voltage (mV), d is the partition thickness (m), Tg is the temperature of specimen surface in the heating chamber and Tc is the temperature of specimen surface in the cooling chamber.
- Acoustic insulation capacity—sound absorption coefficient according to PN-EN ISO 10534-2:2003 [49] within the frequency range of 80–5000 Hz.
- Density profile—determined using a laboratory DAX profile measurement gauge by GreCon (Fagus-GreCon Greten GmbH&Co. KG, Alfeld-Hannover, Germany).

Density, compressive strength and the modulus of elasticity were determined on samples of 100 mm × 100 mm. Tests were performed on 10 specimens. Recorded results were analyzed statistically. Statistical calculations were conducted using the Statistica 13.0 software (StatSoft Inc., Tulsa, OK, USA).

## 3. Results and Discussion

Prior to the tests, pastes were prepared from the selected agents and used to bond two beech wood specimens. Specimens were bonded at 120 °C under the pressure of 1.4 N/mm^2^. Next, the specimens were subjected to the joinery test (nonstandardized chisel test) in order to evaluate bond quality. The best results (the largest number of fragments of pulled-out wood) were recorded for starch solutions, while potato starch and rye flour solutions proved to be slightly weaker bonding agents.

Properties of panels manufactured from fibers with the initial moisture content are presented in Table 2. As it results from these data, the recorded densities slightly deviate from the assumed value, which results from the still imprecise determination of increments in dimensions resulting from fitting to the size of the mold in which the mat was formed. Nevertheless, the obtained results can be considered satisfactory.

**Figure 1:**
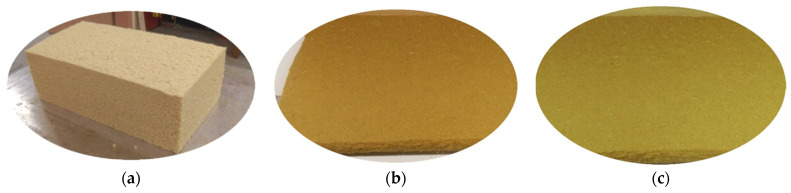
(**a**) The view of the mold before pressing. (**b**,**c**) The view of the board after pressing at (**b**) 180 °C and (**c**) 150 °C.

The view of the mold before and after pressing is shown in Figure 1. Panels after pressing, apart from the slight changes in length and width in relation to the assumed values, slightly darkened under the influence of applied temperatures. As it results from the data given in Table 2, compressive strength of panels resinated with UF adhesive is 99.7 and 515.7 kPa for boards with the density of 175 and 240 kg/m^3^, respectively. Thus, in their case, an increase in density by slightly below 40% causes an increase in strength by over 500%. In contrast, all of the panels manufactured using potato starch and rye flour exhibit much lower compressive strength. The greatest compressive strength was recorded for panels produced with a 12% share of rye flour. This is approx. 20% lower than the value for panels manufactured from wood fibers resinated with UF adhesive. A decrease in the bonding rate with the rye flour solution from 12% to 6% leads to a reduction in compressive strength by almost a half. The compressive strength of panels manufactured from wood fibers bonded with the potato starch solution is generally much lower than that of panels manufactured with a share of rye flour.

**Figure 2:**
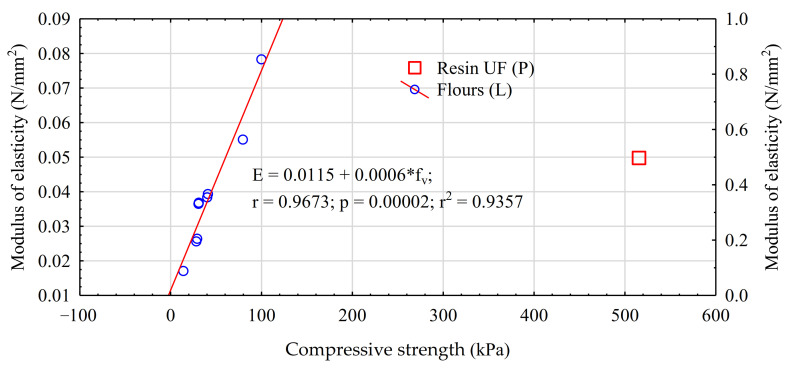
Correlation of modulus (E) of elasticity with compressive strength (f_v_).

Strength properties to a considerable degree depend on panel manufacturing conditions. Considerably better results are obtained when applying longer pressing times and lower heat press platen temperatures rather than vice versa. Unfortunately, at a very long pressing time of panels at 180 °C, a very strong darkening of the panel surface is observed, resulting from fiber degradation. The use of potato starch solutions is problematic, since their dry mass is very low; thus, in order to obtain a minimal required bonding rate (the ratio of the dry mass of the active component to the dry mass of fibers), a very large amount of the solution needs to be applied. Although panel density was reduced to accelerate water vapor evacuation, this is a relatively long process. However, the obtained compressive strength levels for these variants do not have to be considered as very negative, as in terms of the bonding rate the obtained results were comparable or even more advantageous than those recorded for the variant with the application of UF resin. This is obviously assuming the linear dependence of strength on resination rate, which may not be considered as certain. Table 3 also gives results of the analysis of homogeneity for the groups determined using the LSD test. However, at very large differences in compressive strength between individual panel types, this analysis is not highly informative. The modulus of elasticity for panels manufactured using natural adhesives is lower than that of panels produced using urea–formaldehyde resin (Figure 2). This is determined both by the greater density of panels produced using UF resin and the greater stiffness of the glue line itself. A linear correlation between the modulus of elasticity and compressive strength was found for panels manufactured with a share of natural adhesives. Thus, an increase in the level of this property may be expected as the panel density increases.

The obtained values of thermal conductivity range from 0.037 to 0.039 W/(m·K), and it is rather difficult to assess the effect of applied modifications. Since these values are consistent with the reports of manufacturers of insulation boards produced from pulp, it needs to be assumed that this parameter is more strongly dependent on fiber quality and panel density, rather than the applied fiber bonding agent.

Since satisfactory results were obtained for physicomechanical properties when applying relatively long pressing times, it was decided to reduce the amount of water introduced to the system. For this purpose, panels were again manufactured from pine fibers under laboratory conditions; however, this time it was decided not to introduce the adhesive in the liquid form. Gelatinized starch and oxidized starch (E1404) were used as bonding agents. When the adhesive is applied in the powder form, it is relatively easy to modify the amount used, and the level of mat moisture content before pressing is much more advantageous. Other tests, not described in this paper, were also performed, in which starch was applied on dry fibers and moisture content was increased to reach 12–15%. However, it was observed that when employing such a method of starch application, some of the grains are left in the mixer. Data in Table 3 indicate that this method also provides satisfactory results, while it is easier to apply and facilitates a considerable reduction in panel manufacturing time. The obtained compressive strength levels were comparable to those of panels manufactured from fibers bonded with urea resin. The most advantageous results were observed for a 20% share of starch in relation to pulp and pressing time of 18 s per 1 mm panel thickness. Panel density was increased so that their results could be compared to commercial panels. As it results from the values given in Table 2, wet-formed panels of such density exhibit strength lower than that of laboratory panels by as much as 50%, and it may be assumed that it is exactly 50% lower than that of dry-formed boards. It may be inferred that a small amount of phenol–formaldehyde resin was used in the former case and pMDI was used in the latter case.

**Figure 3:**
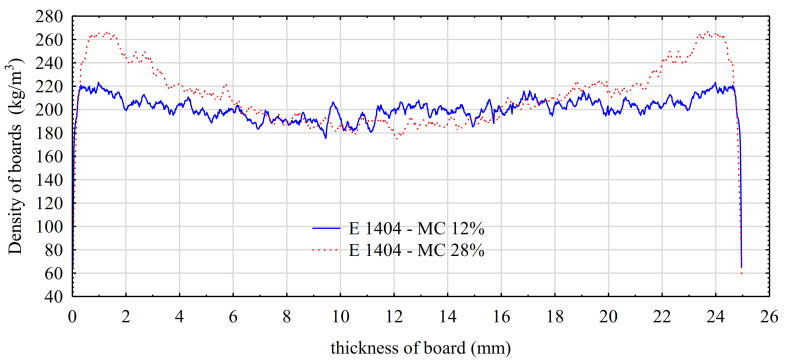
Density profiles of boards manufactured from pulp differing in initial moisture content.

A change in the method of fiber bonding agent application has no effect on the level of thermal conductivity. In this case, the value of λ was within the range of 0.037–0.038 W/m·K, and no influence of the applied board manufacture parameters was observed. In turn, the level of moisture content in the pressed mat has a significant effect on the density profile of manufactured panels. As it results from values presented in Figure 3, boards manufactured from pulp with an approx. 28% moisture content have considerably more compacted subsurface zones. In that area, a density increase to over 260 kg/m^3^ was recorded in the case of panels manufactured from pulp with a greater moisture content. In turn, for pulp with an approx. 12% moisture content in the subsurface zone, the density of subsurface layers slightly exceeds 220 kg/m^3^ and is only slightly higher than in certain areas located deeper. This dependence may explain such advantageous properties of *E0* panels at compression perpendicular to grain despite their low initial moisture content. Thanks to the more uniformly distributed density at the cross-section, they effectively transfer external loads.

**Figure 4:**
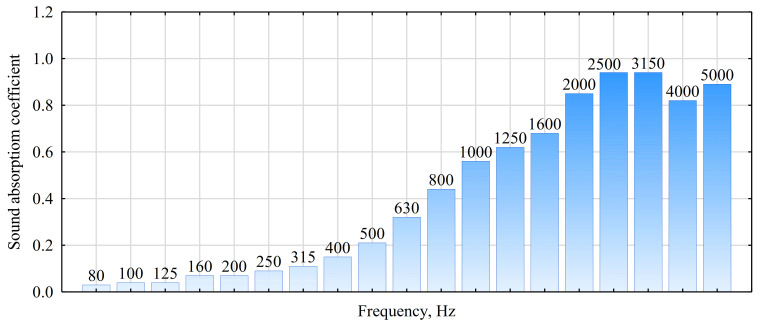
Absorption coefficient of fiberboard glued with E1404.

Panel E0 was selected for tests assessing the sound absorption coefficient. Although such panels did not exhibit the highest strength parameters, their manufacturing process ultimately seems the easiest to perform, since the fibers used to form the mat contain the lowest amounts of water. Figure 4 presents a graph for sound absorption of the analyzed panel. The most advantageous behavior is observed for this panel for frequencies over 2000 Hz, although it may also be stated that advantageous values of the sound absorption coefficient are obtained in the frequency range of 500 to 1600 Hz. It is difficult to assess the effect of the bonding agent itself on sound absorption, since the recorded values are very similar to those for conventional lightweight insulation fiberboards or panels from natural fibers [50,51].

## 4. Conclusions

These tests are preliminary studies aiming at the identification of easy-to-use, readily available compounds that exhibit bonding properties in contact with water. Although a wide range of such products has not yet been launched on the market, numerous similar studies indicate considerable potential applicability, particularly for these agents, which are food components. This is because they are easily processed and are fully biodegradable, which in combination with wood makes it possible to manufacture products completely neutral in terms of their environmental impact. It seems that the trend towards the application of such agents to produce insulation materials is promising, since such panels are not required to exhibit high mechanical strength; as a result, meeting the relevant requirements is easier. On the other hand, lightweight panels of very low density are more difficult to produce because they fail to mask errors in the bonding and forming technology in terms of uniformity of adhesive application and variable density. Nevertheless, such production processes are deserving of further investigation, which may be aided by the following results from this study:

- In the application of food components as bonding agents in the manufacturing process of lightweight insulation panels from wood fibers, the obtained properties may be comparable to those of commercial panels produced using synthetic agents.
- The compressive strength of manufactured panels to a considerable extent depends on the ratio of the dry mass of the active agent to the dry mass of fibers.
- Modification of the bonding rate is dependent on the feasibility of preparation of a solution with a specific concentration from a given flour or starch.
- Since low concentration solutions are required, pressing time needs to be extended due to the considerable increase in moisture content of the formed pulp.
- It seems that although this problem has not been fully solved, the use of starch in the powder form on fibers with a high moisture content has considerable potential applicability.

## Figures and Tables

**Table 1:** Pressing variants applied.

Bonding Agent	MC Fiber, %	Symbol	Density of Board, kg/m^3^	Bonding Rate, %	Temperature, °C	Pressing Time, s/mm of Thickness
UF	7.2	U1	175	12	180	30
		U2	250	12	180	30
Rye flour	7.2	R1	175	12	180	34
		R2	175	6	180	34
Potato starch	7.2	P1	150	2.4	180	38
		P2	150	3.6	180	38
		P3	150	2.4	150	38
		P4	150	3.6	150	38
		P5	150	2.4	150	48
		P6	150	3.6	150	48
E 1404	12	E0	200	12	180	18
Gelatinized starch	28	S1	200	12	180	24
Gelatinized starch		S2	200	20	180	18
Gelatinized starch		S3	200	20	180	12
E 1404		E1	200	12	180	24
E 1404		E2	200	20	180	18
E 1404		E3	200	20	180	12

**Table 2:** Effect of manufacturing parameters on the compressive strength of manufactured boards—fiber moisture 7.2%.

Bonding Agent	Symbol	Density of Board, kg/m^3^	Density of Board, kg/m^3^	Compressive Strength, kPa	Coefficient of Variation, %
UF	U1	175	99.7 ^e,^*	14.3	0.037 (±0.001)
	U2	240	515.7 ^f^	9.97	0.039 (±0.001)
rye flour	R1	174	79.6 ^d^	3.79	0.038 (±0.001)
	R2	165	40.1 ^c^	6.14	0.037 (±0.001)
potato flour	P1	142	29.2 ^b^	10.3	0.037 (±0.001)
	P2	144	28.3 ^b^	28.6	0.037 (±0.001)
	P3	137	14.0 ^a^	7.86	0.037 (±0.001)
	P4	153	30.9 ^b^	13.1	0.038 (±0.001)
	P5	156	30.6 ^b^	11.3	0.038 (±0.001)
	P6	159	40.8 ^c^	22.6	0.039 (±0.001)

* Letters a–f mean homogeneous groups for the NIR test.

**Table 3:** Effect of manufacturing parameters on the compressive strength of manufactured boards—fiber moisture 28%.

Bonding Agent	Symbol	Density of Board, kg/m^3^	Compressive Strength, kPa	Coefficient of Variation, %	Thermal Conductivity W/(m·K)
Control board *	-	201	55.5 ^a^	4.88	-
Control board **	-	200	110	-	-
E 1404	E0	203	88.6 ^b^	9.2	-
Gelatinized starch	S1	189	85.1 ^b^	14.6	0.038 (±0.001)
Gelatinized starch	S2	209	117.0 ^d^	16.1	0.038 (±0.001)
Gelatinized starch	S3	206	95.6 ^b,c^	10.4	0.037 (±0.001)
E 1404	E1	199	96.8 ^b,c^	2.63	0.038 (±0.001)
E 1404	E2	216	133.0 ^e^	13.4	0.038 (±0.001)
E 1404	E3	200	89.9 ^b^	10.6	0.038 (±0.001)

* Control board 1—an industrial wet-formed fiberboard, covered with an additional agent to increase surface water resistance. ** Control board 2—an industrial dry-formed board glued with synthetic agents, the values for which were determined on the basis of commercial information. Letters a–f mean homogeneous groups for the NIR test.

## Data Availability

The data presented in this study are available on request from the corresponding author.
